# How well do policymakers address stigma surrounding substance use disorders: lessons from a qualitative review of Scottish Alcohol and Drug Partnerships’ strategic plans

**DOI:** 10.3389/fpubh.2023.1209958

**Published:** 2023-06-30

**Authors:** Robin Falconer, Jason Tang

**Affiliations:** ^1^Faculty of Health Sciences and Sport, University of Stirling, Stirling, United Kingdom; ^2^Nursing, Midwifery and Allied Health Professions Research Unit, University of Stirling, Stirling, United Kingdom

**Keywords:** substance use, stigma, evidence-informed policy, policy development, strategic planning

## Abstract

**Background:**

Stigma is a significant barrier to the successful implementation of public health policies which aim to reduce harm from substance use disorders. Despite attention being given to stigma in the literature for at least a decade, evidence on what works to reduce it is limited and inconclusive. Without clear guidance, policymakers could be limited in their ability to develop evidence-informed strategies for reducing stigma. In response to a steep incline in drug-related deaths in Scotland since 1996, the Scottish Government has committed to tackling stigma in national drug policy. Scotland’s 31 Alcohol and Drug Partnerships are responsible for developing local strategies that aim to tackle harm from substance use disorders. This qualitative review explored how well these strategies respond to stigma and identified approaches mentioned that could have implicit implications for tackling stigma.

**Methods:**

The strategic plans of Alcohol and Drug Partnerships across Scotland were identified and thematically analysed to identify key themes relating to stigma. Content of strategic plans was initially coded under a coding scheme of four broad categories: content that explicitly mentioned stigma; identity, status and power; deservedness of support; and attribution of responsibility for SUDs.

**Results:**

Twenty-four strategic plans were identified and analysed, with four themes emerging: (1) limited clarity and consistency on how stigma will be directly tackled by ADPs; (2) recognition of the positive contribution that people with substance use disorders can make towards decisions about treatment and support; (3) diversion of people with substance use disorders away from the criminal justice system towards quality support underpinned by human rights; and (4) recognition of the complex determinants of substance use disorders and that everyone has a role to play.

**Conclusion:**

Alcohol and Drug Partnerships acknowledged the importance of tackling stigma in their strategic plans but provide limited clarity on how this will be done. This review calls for the inclusion of more evidence-informed strategies for tackling stigma within the Scottish local policymaking context. This requires academic, policymaking and lived experience communities to collaborate to test and evaluate innovative responses to tackling in stigma to strengthen understanding of what works in which contexts.

## 1. Introduction

### 1.1. Burden from substance use disorders and the Scottish policy response

Substance use disorders (SUDs) were estimated to have affected over 38 million people globally in 2020, representing an increase of 2 million compared to 2019 (1). Drug use, specifically, has been linked to 11.8 million deaths annually (2) with 0.5 million of these involving illicit drugs (3). The number of people using drugs has increased by 22% in the last decade and is projected to further increase by 11% by 2030 (4).

In Scotland, there has been an upward trend in drug-related deaths since 1996, reaching an all-time high of 1,339 deaths in 2020 (5). While recent figures have shown a reduction of 9 (1%) drug-related deaths in 2021 compared to the same period of 2020, it is too early to predict whether the trend is reversing (6). Scotland has consistently recorded the highest drug-related mortality rate in Europe for the past decade (7). In 2018, for example, it recorded 295 deaths per million adult population aged 15 to 64, a record that was significantly higher than Sweden which had the second highest rate of 81.5 deaths per million (7). Such figures underline the urgency for clear policy responses that can serve to mitigate further harm from SUDs.

The Scottish Government’s strategy for reducing harm from drugs and alcohol - *Rights, Respect and Recovery* - has advocated for a ‘human rights-based, public health approach’ to SUDs (8, p. 2), highlighting stigma as a significant barrier to support. The Scottish Drug Deaths Taskforce subsequently published *A Strategy to Address the Stigmatisation of People and Communities Affected by Drug Use* (9) to inform actions for reducing stigma. This recent focus on stigma appears to be a paradigm shift from the previous national drug strategy, *The Road to Recovery* (10), which made only a single reference to stigma in relation to protecting ‘children affected by their parents’ substance misuse’ (p. 50).

### 1.2. What is stigma and why is it important to address in relation to SUDs?

Stigma is a complex social phenomenon, and because of its ubiquitous use, it can be perceived as a loosely defined term that lacks clear conceptualisation (11). Early work by Erving Goffman articulated stigma as a discredited attribute possessed by an individual which becomes a defining feature of that person’s social identity (12). It is often based on assumptions about the stigmatised characteristic, regardless of stigmatised individuals’ behaviours, and is therefore linked to stereotypes, prejudice and discrimination (13). Yang et al. identified three domains of stigma: stereotyping, general emotional reactions, and status loss and discrimination (14). For example, people with substance use disorders (PWSUDs) may be perceived as dangerous, which triggers fear, and subsequently leads to avoidance or degrading treatment of PWSUDs. Stigma also involves the exertion of power to keep people down (oppression), in (sustain norms) or away (disease avoidance) (15) and can be intersectional, in that individuals can possess multiple stigmatised attributes, such as having a SUD as well as a mental health problem (16). It has also been considered a multi-level phenomenon, operating at individual, interpersonal, community and structural levels (17), thus interventions for tackling stigma should consider targeting each of these levels in their design.

The impact of stigma has been studied in relation to a wide range of health conditions, including mental health problems, bloodborne viruses, obesity and cancer (18). However, SUDs are among the most stigmatised conditions, largely resulting from a perception that PWSUDs are to blame for their situation (19). Stigma creates barriers to support, employment, and social integration (14) and can also result in fewer resources being allocated by policymakers to support PWSUDs (13). Collectively, these barriers contribute towards the continued entrenchment of SUDs in society (14).

### 1.3. What will this review add?

A range of approaches for tackling stigma have been proposed in the literature, including group therapy, motivational interviewing, educational programmes, protest and creating opportunities for positive social contact between people with SUD and the wider public (20–22). However, findings from systematic reviews of the utility of such approaches for reducing stigma by Livingston et al. and Tostes et al. were inconclusive due to significant heterogeneity in study designs, target audiences, measures of effectiveness, and lack of longitudinal studies (20, 21). Both recommended that interventions, beyond those included in their reviews, are developed and evaluated. Moreover, in their review of multi-level interventions, Rao et al. highlighted the need for further research to evaluate interventions operating at multiple levels given that stigma is a multi-level construct (17). The limitations in understanding what works to reduce stigma presents challenges for policymakers when deciding how to allocate their resources most efficiently to tackle stigma. Our review, therefore, assessed how this inconclusive evidence-base impacted on policymakers’ ability to articulate clear strategies for tackling stigma.

In Scotland, 31 Alcohol and Drug Partnerships (ADPs) are responsible for developing strategic plans (SPs) to tackle drug and alcohol harms at a local level (23). However, the extent to which these SPs respond to stigma is unknown. Our review sought to: (1) identify actions explicitly focused on addressing stigma in SPs; (2) determine the extent to which these were informed by evidence; and (3) identify any broader strategic approaches included that could have indirect implications for tackling stigma.

## 2. Methods

### 2.1. Search strategy

The lead author conducted a search of relevant local websites in December 2021 to identify the overarching SP for every ADP in Scotland. Websites included those of Health and Social Care Partnerships, Health Boards, Local Authorities as well as ADP websites where these existed. A Google search was used to help locate these websites and to identify SPs. Key search terms *alcohol and drug(s)* in combination with *strategy*, *strategic plan or strategic framework* and the name of each ADP, retrieved from a contact list of ADPs on the Scottish Government website,^1^ were entered into the search (please see Supplementary file 1 for full search strategy).

An e-mail request for the relevant document was sent to the lead officers of any ADPs for which the SP could not be located online. An online search was used rather than contacting lead officers from the outset so that the feasibility of conducting a local policy review via publicly available sources could also be tested and used to inform future reviews. A similar approach was followed by Just et al. in their review of national policies (24).

### 2.2. Eligibility

An assumption was made that each ADP would have only one overarching strategic plan in place at any given time. Therefore, the eligibility criteria acted as a guide to identify the relevant SP for each ADP.

#### 2.2.1. Inclusion

Documents were included if they: (1) contained the key search terms in the title; (2) stated the name of the ADP on the front page; (3) stated the date range the SP applied to.

#### 2.2.2. Exclusion

Expired SPs were excluded. These were determined by an end date stated that was prior to the year the search was carried out.

### 2.3. Screening

Screening was an iterative process during the search. The lead author screened the titles of documents featured on the first page and on subsequent pages if search results continued to be relevant to attempt to identify the document meeting the eligibility criteria for each ADP. For documents received by e-mail from lead officers, titles were screened to check their eligibility. Where there was uncertainty about the eligibility of a document, this was discussed with the second author and agreement reached on whether it should be included.

### 2.4. Analysis

Thematic analysis was selected as the methodological approach for this study. Eligible documents were uploaded to NVivo (QSR International Pty Ltd. Version 12, 2019) which was used to thematically analyse the data. This was informed by Braun and Clarke’s six-stage thematic analysis (25) which involved: familiarisation with the content of documents, coding relevant sections of text, generating initial themes, reviewing themes, defining the themes, and writing up the results.

The analysis was informed by the authors’ theoretical understanding of stigma and content was initially coded using a coding scheme of four broad categories that reflected this: content that explicitly mentioned stigma; identity, status and power; deservedness of support; and attribution of responsibility for SUDs.

## 3. Results

It was expected that 31 SPs would be located - one for each ADP. However, six could not be located as they were unavailable online and no response was received from the lead officers following an e-mail request and one follow-up reminder. Four documents did not contain all key search terms in their titles; however, as the lead officers confirmed that these constituted their SPs and they followed a broadly similar format to the other documents, the authors deemed it relevant to include them. Of the 25 that were located, one was excluded as it was published in the format of a series of webpages, and it was not possible to determine the content that constituted the strategic plan. Therefore, 24 were included in the analysis. The period that SPs covered varied significantly, ranging from one to 11 years (see Supplementary file 2 for a full record of strategic plans).

The four categories used as the coding scheme to organise the content within SPs also allowed identification of included actions that were implicitly or explicitly related to stigma. From the four categories used as the coding scheme to organise content, these were refined further to reflect what was specifically found within the documents.

### 3.1. Theme 1: limited clarity and consistency on how stigma will be directly tackled by ADPs

This theme emerged from the content of SPs that explicitly mentioned stigma (*n* = 20). Of those that did, most statements made were broad and focused on why tackling stigma was important and the perceived sources of stigma, for example:

“…stigma can exacerbate social isolation with consequences for the health and well-being of individuals and communities. Tackling stigma is vital for improving outcomes and reducing drug-related deaths.” (SP 9)

Six SPs described stigma as a barrier to support. One of these stated that it was a greater barrier for women but did not explain the reasons for this. A few SPs also highlighted the need to tackle stigma at multiple levels, as illustrated by the following quote:

“There are three levels of stigma which need to be addressed: societal stigma, institutional stigma, self-stigma/stigma by association.” (SP 17)

While SPs acknowledged the need to tackle stigma, only 13 contained details of specific actions that would be taken to do this. However, due to the small number of SPs that mentioned each action, the authors did not assign these to unique sub-themes. Actions related to education and training were mentioned in the most SPs (*n* = 7). The target audience for these were staff, including health, social care and pharmacy staff. Targeting people identified by PWSUDs as having previously displayed stigmatising attitudes was mentioned in one SP, but it did not state who these were. One SP advocated a broader approach, stating that there should be ‘education for everyone’ (SP 4). SPs did not detail the intended content of education or training nor how it was intended to work to address stigma. The following is an example of the broad statements made in SPs.

“Education and training around stigma is developed and rolled out to staff.” (SP 4)

Six SPs advocated for increasing the visibility of recovery but only one indicated how this would work to reduce stigma, as follows:

“Increasing the visibility of recovery helps reduce stigma and can put a human face to the complex issues underlying drug and alcohol use.” (SP 1)

One SP stated that ‘printed and social media can perpetuate stigma while there is little reporting on positive recovery’ (SP 5), suggesting that increasing the visibility of recovery aims to counteract this. Two ADPs broadly outlined how they would increase the visibility of recovery, stating in their SPs that they would promote positive stories and images.

Campaigns were mentioned in three SPs as an approach for reducing stigma. One of these focused on ‘challeng[ing] use of stigmatising language’ (SP 9) but the others did not specify a focus, as illustrated by the following quote:

“We will campaign to challenge and reduce stigma faced by problematic substance users and their families so that they can move forwards in their recovery to lead safe, healthy and meaningful lives as members of our community.” (SP 23)

A few ADPs mentioned ways that they would monitor the impact of actions to reduce stigma with some SPs listing broad outcome statements, for example:

“Individuals, families and communities affected by alcohol and other drugs will perceive more kindness, compassion and respect, less prejudice and stigma…” (SP 2)

Others outlined specific ways they would measure progress, such as the number of people completing training sessions and undertaking surveys of people’s experiences of stigma, however, it was unclear how some indicators related to stigma. For example ‘rating of neighbourhood by SIMD (Scottish Index of Multiple Deprivation) – gap between 1st and 5th quintile’ and ‘child poverty rates nationally’ (SP 3).

Two SPs suggested that local research would be used to develop further understanding of stigma and to inform actions, for example:

“A…Public Attitudes and Solutions survey…will be used inform local developments to address stigma…” (SP 9)

While this theme was linked to explicit statements about stigma in SPs, the authors also identified several themes that they perceived to have implicit implications for stigma. These were informed by their theoretical understanding of stigma, particularly in relation to the concepts of identity, power and attribution of responsibility. These are outlined below.

### 3.2. Theme 2: recognition of the positive contribution that PWSUDs can make towards decisions about treatment and support

This theme was developed from the content of SPs that the authors perceived to relate to the identity, status and power of PWSUDs. All SPs acknowledged the positive contribution that PWSUDs can make towards decisions about treatment and support. Two sub-themes were identified to reflect distinct levels of decision making.

#### 3.2.1. Person-centred support

Most SPs advocated for a person-centred approach to treatment and support for SUDs. The following quote is an example of the statements made to this effect:

“Person-centred approaches will be developed across treatment and recovery services and the range of health and social care services which work with people with alcohol and drug problems.” (SP 1)

However, very few SPs explained what person-centred support looked like in practice. For those that did, it was about building on people’s strengths, offering ‘maximum choice’ (SP 11), ‘ensuring people using services are in control of their own treatment and support’ (SP 6), and services ‘evolv[ing] to the needs of individuals, rather than the other way round’ (SP 20).

Several SPs also mentioned the need for services to address ‘broader health, care and social needs’ (SP 1, SP 20, SP 30). Another elaborated further, stating:

“Support the person not just the condition: your support/treatment should consider key issues affecting your life as well as supporting you to manage your condition.” (SP 19)

However, no SPs outlined specific ways that ADPs would implement person-centred approaches.

#### 3.2.2. Valuing lived experience

Consistent across all SPs, ADPs also highlighted the importance of involving people with lived experience (PWLE) in wider decision-making processes, including the design, delivery, and evaluation of support.

However, across SPs there were slight differences in how lived experience was defined. For some ADPs, lived experience included other people closely associated with PWSUDs, as illustrated by the following quote:

“The ADP defines lived experience as a person using substances, in recovery, or with previous experiences of drug or alcohol use as well as a person with current or previous experience supporting/caring for someone in recovery or being impacted by someone else’s substance use.” (SP 6)

For other ADPs, family members are carers were mentioned separately from PWLE, for instance:

“Involve people with lived and living experience, families and carers in all aspects of the planning, delivery and evaluation of drug and alcohol service provision.” (SP 4)

There were also differences in how SPs described the extent to which PWLE would be involved. For some, the focus was on listening to, learning from and receiving feedback from PWLE whereas a few others considered PWLE to be ‘key experts’ (SP 1, SP 28), ‘equal members of the ADP’ (SP 4) and an ‘active partner’ (SP 3, SP 24), advocating for a more collaborative approach. Some ADPs had created positions for PWLE on the ADP itself. For example, one SP stated:

“We have established opportunities for a number [of] people with lived and living experience to become full and equal members of the ADP.” (SP 4)

However, one ADP described having not been able to fill these positions and stated that PWLE ‘may recommend an alternative approach to representation’ (SP 9). Another ADP advocated a more independent approach, stating that PWLE should be ‘supported to organise and develop collective and individual voices’ (SP 4).

Several ADPs had developed peer support roles through which PWLE were involved in the direct delivery of support to other people affected by SUDs. One indicated that it had already implemented ‘a pathway from user of services to peer worker to other professional roles and all positions…are recruited in ways which minimise stigma and maximise opportunities for suitable candidates with lived experience to be employed’ (SP 6).

Few SPs explained ADPs’ reasons for involving PWLE. Those that did, put forth reasons including understanding the most helpful, valuable and effective ways to support people and to improve services; providing encouragement and hope for recovery; and supporting people to participate in and contribute to their community. Several SPs stated that involvement should be meaningful, but some ADPs described having experienced challenges with this, as the following quotes illustrate:

“This is an area of acknowledged weakness at strategic level; although many pieces of individual development achieve a genuinely effective level of co−production, much decision making excludes those most affected and most expert.” (SP 6)

“We have not found a consistent, regular and meaningful way…to have the voices of people with lived experience influencing the work of the ADP.” (SP 5)

One SP suggested that for PWLE to be meaningfully involved in decisions about service development, professionals and decision-makers needed to ‘hand over power to make changes’ (SP 17); however, it did not explain how this would be done.

### 3.3. Theme 3: diversion of PWSUDs away from the criminal justice system towards quality support underpinned by human rights

This theme was informed by content of SPs that reflected how PWSUDs deserved to be treated by services. Almost all SPs advocated for PWSUDs to be diverted away from the criminal justice system. For example, one SP stated:

“We will maximise the opportunity for diversion from the justice system at every step of the community justice pathway.” (SP 2)

Several SPs used the term ‘vulnerable people’ to refer to PWSUDs in the context of the criminal justice system, suggesting that ADPs view this group as people in need of support, rather than punishment, for example:

“…services should focus on diverting vulnerable people away from the justice system and into treatment and support.” (SP 17)

One SP highlighted that this was important because ‘the criminalisation of this group of people…can increase the risk of harm and premature death’ (SP 17). However, no SP explicitly stated that diverting people from the criminal justice system would reduce stigma.

Another SP stated that ‘the ADP does not take the lead when it comes to a Public Health Approach in Justice’ (SP 9). The role of ADPs appeared to be about ensuring availability of support for PWSUDs as alternatives to the criminal justice system, as alluded to in the following quotes:

“Develop clear pathways for individuals being liberated from prison, ensuring access to services…is timely.” (SP 28)

“Local alternatives to prosecution including therapeutic services, diversionary activities, educational support and employment opportunities are developed…” (SP 23)

The perspective that PWSUDs should be fully supported was corroborated by several SPs that suggested PWSUDs were entitled to have their human rights recognised. One SP suggested that reducing stigma was an important component of this, it stated:

“Individuals, families and communities affected by…drugs will perceive more kindness, compassion and respect, less prejudice and stigma, contributing to their human rights being fully safeguarded and a greater sense of inclusion and belonging.” (SP 2)

Most statements made about human rights were broad and did not state what was required to implement this, for example:

“We will work to protect and ensure that people’s human rights are respected.” (SP 2)

Similar values were expressed in relation to the delivery of services which many SPs stated should be delivered in a way which treats people with dignity and compassion, as exemplified by the following statements:

“People who use health and social care services have…their dignity respected.” (SP 19)

“…we (the ADP) will go out of our way to show kindness, compassion and respect.” (SP 2)

However, again, it was not explained how this would be ensured. Some ADPs hinted that it was about a competent workforce with the right attitudes and values. For example, one SP stated:

“[services should be delivered] by workers who have the right attitudes, values, training and supervision.” (SP 1)

Overall, this theme suggests that ADPs are seeking to ensure PWSUDs are treated with kindness and support, rather than punishment.

### 3.4. Theme 4: recognition of the complex determinants of SUDs and that everyone has a role to play

The final theme related to the content of SPs that reflected who ADPs implied was responsible for addressing SUDs. Most SPs highlighted that addressing SUDs was complex and identified a range of factors that they considered as determinants of SUDs. These factors included health inequalities, poverty and trauma, as illustrated by the following quote:

“…those experiencing problem alcohol and drug use are often affected by other vulnerabilities (including poor mental health, violence against women, poverty, inequalities and health challenges often stemming from trauma…).” (SP 9)

To respond to these, SPs mainly suggested that services needed to be ‘trauma-informed’ but did not articulate how this would be done. One SP pointed out training as the main approach, stating the following:

“Our focus will be on ensuring key workforce groups are trained to understand the impact of trauma and to deliver their practice in a manner that is trauma informed.” (SP 9)

Others described the need for increased partnership working, for example:

“Recognising and responding to this complexity requires a more collaborative way of working.” (SP 2)

It was not only services that SPs suggested had a role to play in addressing the determinants of SUDs. For example, all SPs highlighted the importance of supportive communities. Communities were considered by many ADPs to play an important role in supporting people’s broader health and wellbeing needs, as the following quote illustrates:

“Working with our communities, recognising the valuable role that people have in supporting themselves to stay well and supporting each other when care is needed.” (SP 1)

However, how ADPs defined communities was unclear. The authors’ assumption was that it broadly referred to the places and social networks that people were part of, out with formal services. For example, one SP highlighted communities as one of several key stakeholders:

“The [ADP] exists to bring people together; people with lived experience, communities, statutory bodies, community groups, voluntary organisations, community planning partnerships, public bodies and health and care providers.” (SP 2)

The term ‘recovery communities’ was also notably used by almost all ADPs in their SPs. However, again, it was unclear what constituted a recovery community and whether they were distinct from wider community support. The following is an example of a statement made about recovery communities:

“We will continue to support the development of our network of recovery communities.” (SP 2)

While SPs acknowledged the importance of supportive communities, very few outlined specific actions to achieve this. A few SPs suggested that ADPs had a responsibility to ensure that communities were supportive. For example, one SP stated that the ADP would ‘develop meaningful community connections and relationships with people to promote better inclusion, health and wellbeing and to reduce social isolation’ (SP 1). Another SP stated that it was the ADP’s role ‘to ensure that there are appropriate supportive opportunities to allow people to sustain their recovery in their community’ (SP 1). Two ADPs stated in their SPs that they would strengthen pathways into community-based support. A few SPs also specifically mentioned the need to tackle stigma, portraying it as a barrier to community support, as illustrated by the following statement:

“We will work with strategic partners to tackle stigma and inequalities that some communities experience related to alcohol and other drugs and improve equality of access to support.” (SP 2)

One SP described the need for PWSUDs to be part of supportive communities even if they were still using substances, as follows:

“…many opportunities to be active and to join communities have not attracted people until they are in or near abstinence…However, isolation among those who are continuing to…use drugs is a well−established risk factor and a source of much distress.” (SP 6)

Many SPs also indicated that PWSUDs should be supported to have an active role in developing support in their own communities. For example, one SP stated:

“Enable our citizens to have opportunities to maintain their wellbeing and take a full and active role in their local community.” (SP 1)

Although SPs acknowledged the wider determinants of SUDs, most also alluded to an expectation of individual responsibility, outlining actions that focused on supporting individuals to make informed choices. These largely, but not exclusively, related to school-based education, with an emphasis on knowledge and information. This emphasis is illustrated by the following quote:

“We want to ensure that people have access to knowledge and information about drugs and alcohol to encourage personal choice…” (SP 1)

Education was considered by a few ADPs as a ‘whole population approach’, suggesting that the aim of education is to prevent SUDs rather than change behaviours of those with SUDs. Several SPs stated that educational programmes would be evidence-based but the methods of delivery and broad content of these programmes were not outlined in SPs.

Overall, the following quote from one ADP concisely summarises what this theme is about:

“We believe that recovery is possible and that we all have a role to support that.” (SP 3)

## 4. Discussion

This review explored how ADPs directly responded to stigma in their SPs. It also identified actions advocated by ADPs that could have implicit implications for tackling stigma. Overall, most SPs acknowledged the impact of stigma, with some more focused on this than others. Four main themes related to stigma were identified.

### 4.1. Limited clarity and consistency on how stigma will be directly tackled by ADPs

For the first theme, the analysis revealed that most ADPs recognised the importance of tackling stigma, but there was limited clarity within and limited consistency across SPs regarding how this would be done, which could be the result of the lack of consensus in the wider literature on what works to reduce stigma. Although ADPs could have drawn from the publicly available national strategy (9) to inform how they addressed stigma within their SPs, this may not have been possible since the publication of the national strategy and renewal of most ADP SPs coincided during the same year meaning that ADPs may have had insufficient time to incorporate the advice and guidance of the national strategy into their SPs. This highlights the importance of regularly reviewing local SPs to ensure that they are responsive to emerging policy priorities.

Most ADPs advocated three main approaches for tackling stigma: education; campaigns; and increasing the visibility of recovery. Systematic review findings on the effects of education in reducing stigma indicate that education can be effective, but their success is dependent on the target audience, mode of delivery, and measurement of outcomes (20, 21). There is also evidence that such educational approaches can be bolstered by incorporating opportunities for positive social contact between target audiences and PWSUDs (20). In support of such approaches, van Boekel et al. found that health professionals who had more frequent contact with PWSUDs displayed more positive attitudes towards them than those with minimal contact (26). However, not all findings on the utility of education have been positive. For example, in a cross-sectional survey of general nurses’ attitudes towards PWSUDs, Ford et al. found that education could be counterproductive if nurses did not feel supported in their role. This was because education increased role expectations but without role support, nurses felt less confident in their ability to provide care for this patient group (27). Collectively, these studies suggest that education alone is unlikely to be sufficient for reducing stigma and that ADPs should develop and evaluate multi-component interventions. As the SPs we reviewed did not articulate what was meant by education and training and did not explicitly specify the target audience, it was difficult to ascertain the extent to which SPs were evidence informed. As such, SPs could be strengthened by more clearly articulating what they mean by education or training, what precisely this involves, and the target audience.

Some SPs endorsed the use of campaigns for tackling stigma but, again, none were explicit about what constituted a campaign, who these should target and the intended duration. Further, we found limited evidence in the wider literature to suggest that campaigns were effective at reducing stigma surrounding SUDs and there appeared to be an absence of studies to show whether campaigns had any effect on long-term behaviour change (28, 29). Campaigns, however, could involve a range of components as well as delivery modes so it is crucial that ADPs specify what they mean by this term so that it can be evaluated through empirical studies and to allow for its replication in different contexts if found to be effective. Walsh and Foster warned against use of simplified campaign messaging that attempts to change attitudes through addressing a perceived knowledge-deficit, asserting that messages which encouraged the public to view SUDs as a health condition could reinforce the belief that SUDs only happen to certain people (30). Thus, ADPs should ensure that they specify the target audience for campaigns and that they take into account underlying reasons for stigmatising beliefs held by these audiences so that campaign resources can be targeted effectively and efficiently (31).

With regards to increasing the visibility of recovery mentioned in a few SPs, the evidence indicates that this approach can be successful in reducing stigma through a range of delivery methods. In a randomised controlled trial study, leaflets distributed to the public that portray those with SUD positively were found to be more effective at reducing stigmatising attitudes than leaflets including key facts alone (32). Moreover, increasing the visibility of recovery is crucial because recovery is rarely celebrated out with the confines of closed recovery groups and often only the negative consequences of SUDs are visible to the wider community. Best and Colman argued this point cogently, highlighting that such one-dimensional portrayals of PWSUDs can increase resentments towards this group, further exacerbating stigma (33). They also further argued that exclusion of PWSUDs from communities breaks down community bonding. Thus, ADPs should consider within their SPs the inclusion of approaches designed to increase public awareness of the community benefits of reducing stigma towards PWSUDs.

### 4.2. Recognition of the positive contribution that PWSUDs can make towards decisions about treatment and support

It was evident that ADPs recognised the positive contribution that PWSUDs can make towards decisions about their own support as well as wider strategic decision-making processes. Most SPs mentioned person-centred support and portrayed this as a way of providing more individual choice and control over the type of treatment and support they receive. This might be difficult to achieve in practice given that within mental health services, self-stigma has been known to reduce willingness to question or speak openly with health professionals (34). However, some ADPs mentioned that they had implemented peer and advocacy support services which could minimise self-stigma and, in turn, encourage PWSUDs to engage openly with health professionals about their treatment.

There was also widespread commitment to involving PWSUDs in wider strategic decision-making processes but there was no consensus between ADPs on how this could best be achieved. Given that exertion of power by some groups over others has been identified as a core component of stigma (15), we advise that once an approach has been established which enables meaningful involvement of PWSUDs, the role of all stakeholders must be regularly reviewed to ensure that everyone’s views and contributions are equally represented and reflected in decision making. Unequal power dynamics that may arise should also be resolved, for example by ensuring that professionals do not act as gatekeepers to involvement. This does not necessarily mean shifting power away from professionals but, rather, drawing on the collective knowledge and experience of professionals and PWSUDs (35).

We found that many SPs used the phrase ‘involve people with lived experience (PWLE)’ and suggested that this included relatives of PWSUDs. We believe this to be an important consideration because stigma has been found to also affect those who associate with PWSUDs (36) so their perspectives are also of value. However, as with the barriers to person-centred support, stigma may deter PWLE from disclosing their lived experience (37), potentially rendering approaches for increasing involvement of PWLE in strategic decision-making ineffective. Peer and advocacy support already used by some ADPs can help to reduce barriers of such self-stigma but its utility to reduce stigma more widely needs to be further examined. ADPs may also need to challenge their thinking around traditional decision-making structures and seek feedback from PWLE on how they wish to be involved.

### 4.3. Diversion of PWSUDs away from the criminal justice system towards quality support underpinned by human rights

Almost all SPs mentioned that diverting PWSUDs away from the criminal justice system towards quality support underpinned by human rights was a priority. This theme emerged as being relevant to stigma because the criminalisation of SUDs reinforces negative stereotypes of PWSUDs as dangerous and deserving of punishment (38). Within SPs, a move towards the recognition of the human rights of PWSUDs appeared to be an attempt to change this paradigm but it was unclear from the broad statements identified what ADPs considered to be within their control regarding this. This may be explained, in part, by the Scottish system whereby policing responses are informed by a single, national police force that is likely to inform local policing responses to substance use. However, on a positive note, Police Scotland’s *Annual Police Plan 2022/23* (39) states that they will use ‘public health principles’ to respond to substance use-related harm, suggesting increasing support for the inclusion of diversionary approaches across the country.

Most ADPs suggested within their SPs that they could initiate and manage supportive pathways between the police, the wider criminal justice system, health services and community support. This is promising as, according to Stevens et al., diversion from the criminal justice system relies on ‘relationships between policing systems and other agencies, as well as the capacity of healthcare and welfare systems to provide effective treatment and to support social integration’ (40, p.38). Achieving this collaborative way of working, however, can only be successful if there is a commitment to reduce stigma across all stakeholders, which is challenging since many may harbour beliefs that stigma attributed to criminals is necessary as it acts as a deterrent against substance use and so are likely to be supportive of punitive approaches (41). Developing interventions to change public attitudes towards SUDs are therefore fundamental to increasing the support for alternatives to criminal justice responses.

In general, it was not clear within SPs what ADPs meant by diversion from the criminal justice system. Stevens et al. indicated that diversion involved one of three alternatives to criminalisation, alongside depenalisation and decriminalisation (40). Diversion refers to policy or legislative provisions which divert people away from criminal sanctions towards supportive alternatives, such as education or health and social care services (40). Broadly, depenalisation refers to *de facto* policies aimed at reducing criminal sanctions, and decriminalisation is de jure removal of criminal sanctions (40). Diversion can be further broken down into six levels by the Sequential Intercept Model (42), which proposes that the optimal response is preventing contact with the criminal justice system through high-quality, accessible healthcare services and, thereafter, the other intercept levels aim to prevent further progression through the criminal justice system. Such models could be used by ADPs to explicitly outline what is within their direct control regarding diverting PWSUDs from the criminal justice system.

ADPs prioritised human rights-based approaches over criminal justice responses to SUDs, but they did not clarify what this meant in practice. Policies that advocate for the recognition of human rights without articulating how this will be achieved has been described by Barrett et al. as ‘tokenistic use of human rights’ (43, p. 357). ADPs should, therefore, be as explicit as possible on what they mean when they mention a human rights-based approach. This can be achieved by, for example, using the PANEL (participation, accountability, non-discrimination and equality, empowerment, legality) principles (44), a resource developed to ensure that human rights are transferred into practice (see Supplementary file 3).

Overall, this theme exemplifies how difficult it can be for policymakers to select effective approaches to reduce stigma that can be implemented into practice. Decriminalisation could help to reduce stigma by treating SUDs as a public health issue rather than a criminal justice problem (45). This is also acknowledged by the Scottish Drug Deaths Taskforce which highlighted that socially ingrained processes whereby those in positions of authority decide what is best for PWSUDs in terms of their support and treatment exacerbates stigma (9). While de jure decriminalisation is not within the direct control of the Scottish Government, the implementation of a range of *de facto* diversion and depenalisation responses may be feasible and can be used to reduce intersectional stigma, such as PWSUDs also being perceived as criminals (46). However, generating support for such policies will require policymakers to demonstrate that alternatives to criminalisation do not exacerbate social problems, such as anti-social behaviour.

### 4.4. Recognition of the complex determinants of SUDs and that everyone has a role to play

We identified this final theme to be relevant as it considers the wide range of complex factors that lead to SUDs, rather than on prevailing narratives that emphasise individual blame and responsibility. Encouragingly, we found that almost all ADPs acknowledged this, highlighting that individuals have limited direct control over circumstances as poverty, inequalities and trauma. In relation to trauma, our findings revealed that most ADPs advocated for ‘trauma-informed’ practice which aims to ensure that those who deliver services fully understand the risk of triggering trauma in service users who have previously experienced trauma (47). However, apart from one SP which proposed workforce training as a means to implementing trauma-informed practice, no other SPs explained how trauma-informed practice would be implemented and by whom. Since many studies have shown an association between lower socioeconomic position and increased risk of traumatic events (48), a range of interventions to tackle underlying health inequalities, including stigma as a reinforcer of health inequalities (49), is likely to be required, as well as unconscious bias that health professionals may have towards PWSUDs which can lead to behaviours which exacerbate trauma (50).

Alongside the role of services in supporting PWSUDs, ADPs mentioned that communities also had a role to play. However, they were not explicit about how they defined communities nor what constituted supportive communities. This is perhaps due to ‘community’ being a broad term, that can represent an aggregation of social and environmental features. For example, Best and Coleman categorise communities as *geographic*, related to the places PWSUDs live; and *social networks*, related to the day-to-day social groups that people belong to (33). Therefore, it is important that ADPs articulate what they mean by supportive communities so that they can determine their role in facilitating this.

In the wider literature, the role of supportive communities in relation to SUDs has been clearly articulated. For example, the notion of community recovery capital has been posited as the collective resources available within a community that facilitate recovery from SUDs (51). This includes supportive public attitudes, services and interventions for reducing stigma. Best and Coleman developed the concept of an ‘Inclusive City’ which describes a process through which everyone who is part of a geographic community - including community groups, businesses, and people in recovery - unite to build community recovery capital, which benefits the whole community (33). An Inclusive City is underpinned by the CHIME model, originally developed by Leamy et al. (52), to describe important features of the mental health recovery process. CHIME stands for connectedness, hope, identity, meaning and empowerment, and each of these are an important part of the pathway to building community recovery capital (52). ADPs could use each of these domains to assess features within their geographic area of responsibility which contribute towards positive and negative community recovery capital and to identify actions for building supportive communities.

We found that the term ‘recovery communities’ was also used by almost all ADPs to describe an important feature of supportive communities, however, there are many definitions of recovery communities in the literature. It can be described, for example, as a grassroots movement where people with shared lived experience of recovery come together to create a sense of belonging and provide peer support out with the confines of traditional service opening hours (53). However, it is unclear whether self-organisation is a necessary component of a recovery community and how ADPs can support them. Therefore, further research on recovery communities, including their role in tackling stigma would be beneficial. It would also be prudent for ADPs to seek out and engage with recovery communities to explore how they wish to be supported by ADPs. However, caution from ADPs will be required to respect the grassroots nature of recovery communities, for example, by avoiding attempts to formalise them as part of the infrastructure of commissioned ADP services. Regardless of recovery communities’ association with ADPs, they clearly form an important part of broader support for recovery within geographic communities.

We found that ADPs acknowledged the range of determinants of SUDs that were out with the control of individuals, but they also implied, particularly within their recommendations on school-based drug education, that individuals had a level of responsibility to make informed choices regarding their use of substances. However, it is unclear what the effects of school-based drug education are on attitudes towards PWSUDs from the evidence as this largely unexplored. For example, a report titled *‘What works’ in drug education and prevention* (54) reviewed the evidence on the content of school-based drug education programmes to determine which approaches are effective, but it did not examine stigma as a factor. It did, however, warn that clear evidence of effectiveness is required to prevent unintended harmful consequences. Therefore, we advise that designers of school-based drug education programmes carefully consider the content included to prevent unintended stigma towards PWSUDs, such as by avoiding messages which imply that SUDs is largely due to individual choice.

## 5. Limitations

This is the first review of its kind to explore, using a thematic analysis approach, how SPs of Scottish ADPs respond to stigma surrounding SUDs. Although there is little guidance available regarding qualitative reviews of SPs, we followed a systematic approach where possible to ensure that our search identified all currently active ADP SPs in Scotland. We also purposively selected SPs, drawing upon the inside knowledge of the local policymaking landscape of the lead author. Despite this insight, we found it difficult to access many of the SPs online due to the limited search functionality on the websites searched. Moreover, because there are no consistent criteria for what constitutes a SP, we found screening for eligibility of documents to be challenging and had to rely on our interpretation of document titles or confirmation from lead officers. Several SPs also referred to other documents, such as action plans and SPs for other local issues, which were not explored as they were beyond the scope of this review. It is, therefore, possible that the SPs reviewed did not represent the entirety of ADPs’ responses to stigma.

Like all qualitative research, the findings were subjective of the authors’ theoretical understanding of stigma and interpretation of the SPs reviewed. As the intention was to provide an explorative overview of SPs, not to objectively assess quality or effectiveness, we do not consider this to be detrimental to the review aim. Nevertheless, we encourage readers to apply their own critique to the findings. Finally, as the review focused on the Scottish policymaking context, the findings are not necessarily transferable to other countries. However, the findings can be used by policymakers in other contexts to critically review the content of policy documents, such as strategic plans, to determine whether these provide sufficient clarity on how stigma will be tackled at all levels.

## 6. Conclusion

The findings from this review suggest that, despite ADPs identifying stigma as a priority issue in their SPs and many outlining actions to tackle stigma, SPs provided limited clarity on how ADPs would do this and there was inconsistency across SPs with regards to how stigma will be tackled. This is despite the publication of the Scottish Drug Deaths Taskforce strategy for addressing stigma, suggesting that, at the point of writing, this has not yet filtered into ADPs’ SPs. The limited evidence on what works to reduce stigma surrounding SUDs may be a factor which has prevented ADPs from articulating clear approaches for tackling stigma in their SPs and could have wider implications for evidence-informed policymaking to tackle stigma in other contexts. The lack of direction at a local level may prevent stakeholders from understanding their role in tackling stigma. This could limit attention being given to this issue and the ability of ADPs to monitor how effectively their SPs have been implemented.

The specific actions for tackling stigma mentioned in SPs mainly focus on improving knowledge and awareness through education and training, increasing the visibility of recovery, and campaigns. However, the four themes identified by this review highlights the complexity of stigma and the need for similarly complex approaches to tackle it. Our findings point to three broad approaches that should be explored further with regards to the contribution they make toward tackling stigma: recognition of the positive contribution that PWSUDs can make towards decisions about treatment and support; diversion of PWSUDs away from the criminal justice system towards quality support underpinned by human rights; and recognition of the complex determinants of SUDs and that everyone has a role to play. A concerted effort from academic, policymaking and lived experience communities is now required to test innovative responses to stigma and strengthen understanding of what works to reduce stigma surrounding SUDs to support future policymaking and strategic planning processes.

## 7. Recommendations

- To ensure SPs provide clear direction for all stakeholders responsible for implementing actions to tackle stigma, these should more explicitly articulate:
- actions to be taken to tackle stigma, why these actions are deemed appropriate and how these will be implemented – these should be informed by the evidence currently available.
- ADPs’ role in diverting people with SUDs away from the criminal justice system – assisted by the Sequential Intercept Model (42)
- what a human rights-based approach means in practice – assisted by the PANEL principles (44) (see Supplementary file 3)
- actions to be taken to build supportive communities – assisted by the CHIME model (52).
- ADPs should ensure that the content of educational programmes and campaigns to tackle stigma is informed by research on the underlying beliefs of target audiences.
- Peer advocacy support should be tested as an approach to mitigate against self-stigma which can prevent PWSUDs from fully participating in treatment decisions and wider ADP decision-making processes.
- SPs should be regularly updated to take account of emerging evidence and national guidance on tackling stigma.
- The Scottish Government should commission further research to understand the impact of school-based drug education programmes and other population-wide drug awareness interventions on attitudes towards PWSUDs.

## Author contributions

RF and JT designed the study. RF conducted the qualitative analysis supported by JT. RF wrote the first draft of the manuscript. JT provided critical feedback and editing to subsequent drafts. All authors contributed to the article and approved the submitted version.

## Conflict of interest

The authors declare that the research was conducted in the absence of any commercial or financial relationships that could be construed as a potential conflict of interest.

## Publisher’s note

All claims expressed in this article are solely those of the authors and do not necessarily represent those of their affiliated organizations, or those of the publisher, the editors and the reviewers. Any product that may be evaluated in this article, or claim that may be made by its manufacturer, is not guaranteed or endorsed by the publisher.

## Footnotes

^1^ https://www.gov.scot/publications/alcohol-and-drug-partnerships-contacts/

## Supplementary material

SUPPLEMENTARY FILE 1 Record of strategic plans.

SUPPLEMENTARY FILE 2 Search strategy.

SUPPLEMENTARY FILE 3 Panel principles.

## Abbreviations

ADPs: Alcohol and Drug Partnership
PWLE: People with lived experience
PWSUDs: People with substance use disorders
SPs: Strategic plans
SUDs: Substance use disorders
