# Supplementary material for: How well do policymakers address stigma surrounding substance use disorders: lessons from a qualitative review of Scottish Alcohol and Drug Partnerships’ strategic plans
Source: Front Public Health. 2023 Jun 30;11:1209958. doi: 10.3389/fpubh.2023.1209958 (PMC10348887; doi:10.3389/fpubh.2023.1209958)
Supplement: SUPPLEMENTARY FILE 1 — Record of strategic plans. [file Data_Sheet_1.PDF]

Supplementary file 1 - Search strategy

Search terms (Google and ADP website search for strategic plans)

1. ALCOHOL
2. DRUG$
3. **1 AND 2**
4. STRATEGY
5. STRATEGIC PLAN
6. STRATEGIC FRAMEWORK
7. **4 OR 5 OR 6**
8. ABERDEEN CITY
9. ABERDEENSHIRE
10. ANGUS
11. ARGYLL AND BUTE
12. BORDERS
13. CITY OF EDINBURGH
14. CLACKMANNANSHIRE AND STIRLING
15. DUMFRIES AND GALLOWAY
16. DUNDEE CITY
17. EAST AYRSHIRE
18. EAST DUNBARTONSHIRE
19. EAST RENFREWSHIRE
20. FALKIRK
21. FIFE
22. FORTH VALLEY
23. GLASGOW CITY
24. HIGHLAND
25. INVERCLYDE
26. MIDLOTHIAN AND EAST LOTHIAN
27. MORAY
28. NORTH AYRSHIRE
29. NORTH LANARKSHIRE

30. ORKNEY

31. PERTH AND KINROSS

32. RENFREWSHIRE

33. SHETLAND

34. SOUTH AYRSHIRE

35. SOUTH LANARKSHIRE

36. WEST DUNBARTONSHIRE

37. WEST LOTHIAN

38. WESTERN ISLES

39. **8 OR 9 OR 10 OR 11 OR 12 OR 13 OR 14 OR 15 OR 16 OR 17 OR 18 OR 19 OR 20 OR 21 OR 22 OR 23 OR 24 OR 25 OR 26 OR 27 OR 28 OR 29 OR 30 OR 31 OR 32 OR 33 OR 34 OR 35 OR 36 OR 37 OR 38**

40. **3 AND 7 AND 39**
