# Supplementary material for: How well do policymakers address stigma surrounding substance use disorders: lessons from a qualitative review of Scottish Alcohol and Drug Partnerships’ strategic plans
Source: Front Public Health. 2023 Jun 30;11:1209958. doi: 10.3389/fpubh.2023.1209958 (PMC10348887; doi:10.3389/fpubh.2023.1209958)
Supplement: SUPPLEMENTARY FILE 3 — Panel principles. [file Table_2.DOCX]

**Supplementary file 3 – PANEL principles**

The PANEL principles are internationally recognised principles that have been developed to support policymakers to articulate what a human rights-based approach means in practice.

The table below describes each principle and suggests how they could be applied by Alcohol and Drug Partnerships (ADPs) in the context of tackling stigma.

| **Principle** | **Description** | **Suggested action** |
| --- | --- | --- |
| Participation | People should be involved in decisions that affect their rights. | Ensure people with substance use disorders and close family members/carers are involved in all decisions that affect them. This should be done in a meaningful way, fully considering barriers to participation, including stigma. |
| Accountability | There should be monitoring of how people’s rights are being affected, as well as remedies when things go wrong. | Ensure there is a system in place to monitor how the human rights of people with substance use disorders are affected by strategic decisions and the way support is delivered. |
| Non-discrimination and equality | All forms of discrimination must be prohibited, prevented and eliminated. People who face the biggest barriers to realising their rights should be prioritised. | Set out a clear plan to eliminate stigma within all areas of the ADP’s responsibility. |
| Empowerment | Everyone should understand their rights and be fully supported to take part in developing policy and practices which affect their lives. | Support people with substance use disorders to self-organise and take collective action to address issues that negatively impact on their human rights, including further developing recovery communities and ensuring that decision-making structures have an ‘open door’. |
| Legality | Approaches should be grounded in the legal rights that are set out in domestic and international laws. | Ensure people with substance use disorders are aware of their human rights, as set out in legislation, by actively promoting these. |

Further information on the principles and tools to support implementation are available on the Scottish Human Rights Commission website: <https://www.scottishhumanrights.com/projects-and-programmes/human-rights-based-approach/>
